# NeuroD4 converts glioblastoma cells into neuron-like cells through the SLC7A11-GSH-GPX4 antioxidant axis

**DOI:** 10.1038/s41420-023-01595-8

**Published:** 2023-08-15

**Authors:** Hao Wang, Peiqi Zhao, Ying Zhang, Zhen Chen, Han Bao, Wenqi Qian, Jian Wu, Zhenqiu Xing, Xiaowei Hu, Kunlin Jin, Qichuan Zhuge, Jianjing Yang

**Affiliations:** 1 Department of Neurosurgery, The First Affiliated Hospital of Wenzhou Medical University, Wenzhou, 325000 China; 2 Zhejiang Provincial Key Laboratory of Aging and Neurological Disorder Research, The First Affiliated Hospital of Wenzhou Medical University, Wenzhou, 325000 China; 3 Department of Pharmacology and Neuroscience, University of North Texas Health Science Center, Fort Worth, TX 76107 USA

**Keywords:** CNS cancer, Cancer therapy

## Abstract

Cell fate and proliferation ability can be transformed through reprogramming technology. Reprogramming glioblastoma cells into neuron-like cells holds great promise for glioblastoma treatment, as it induces their terminal differentiation. NeuroD4 (Neuronal Differentiation 4) is a crucial transcription factor in neuronal development and has the potential to convert astrocytes into functional neurons. In this study, we exclusively employed NeuroD4 to reprogram glioblastoma cells into neuron-like cells. In vivo, the reprogrammed glioblastoma cells demonstrated terminal differentiation, inhibited proliferation, and exited the cell cycle. Additionally, NeuroD4 virus-infected xenografts exhibited smaller sizes compared to the GFP group, and tumor-bearing mice in the GFP+NeuroD4 group experienced prolonged survival. Mechanistically, NeuroD4 overexpression significantly reduced the expression of SLC7A11 and Glutathione peroxidase 4 (GPX4). The ferroptosis inhibitor ferrostatin-1 effectively blocked the NeuroD4-mediated process of neuron reprogramming in glioblastoma. To summarize, our study demonstrates that NeuroD4 overexpression can reprogram glioblastoma cells into neuron-like cells through the SLC7A11-GSH-GPX4 signaling pathway, thus offering a potential novel therapeutic approach for glioblastoma.

## Introduction

Glioblastoma, the most common and aggressive primary cancer in the central nervous system (CNS), presents a significant therapeutic challenge due to its poor prognosis and resistance to standard treatments [1]. With an annual incidence rate of 3.23 per 100,000 cases, glioblastoma’s occurrence is influenced by factors such as age, gender, and race [2, 3]. The recent definition of glioblastoma describes it as a diffuse astrocytic glioma with specific histological or genetic features [4, 5]. Despite rigorous treatment approaches, including surgery, radiotherapy, and chemotherapy, tumor relapse remains almost inevitable due to glioblastoma’s infiltrative nature and resistance to current therapies [6]. Therefore, the exploration of new treatment options for glioblastoma is crucial.

Cell conversion through reprogramming of genes and transcription factors that regulate cell fate and differentiation has shown promise [7–9]. Among the regulators in this developmental process, basic helix loop helix (bHLH) transcription factors play a significant role, guiding cell fate determination in different lineages across all three embryonic germ layers [10–12]. Overexpression of bHLH transcription factors such as Neurog2 (NGN2) and ASCL1 has demonstrated the ability to transform human glioma cells into neuron-like cells in culture [13, 14]. Among the shared target genes in the neurogenic programs induced by Neurog2 and ASCL1, only NeuroD4 has been shown to induce neuronal reprogramming [15–18]. Notably, Cheng et al. recently reported that ASCL1, acting as a single transcription factor, can transform human glioblastoma cells into terminally differentiated neuron-like cells, thereby inhibiting their aggressive proliferation [19]. Building upon this knowledge, our aim was to investigate the potential of NeuroD4 overexpression in transforming undifferentiated glioblastoma cells into neuron-like cells, arresting their proliferation both in vivo and in vitro.

In this study, we discovered that NeuroD4 alone effectively reprogrammed human glioblastoma cells into non-proliferating neuron-like cells. This conversion resulted in a significant inhibition of tumor growth both in experimental models and cell cultures. Interestingly, we found that successful reprogramming was dependent on the preservation of cell ferroptosis, as inhibiting this process prevented successful reprogramming. Furthermore, NeuroD4 overexpression significantly reduced the levels of SLC7A11 and Glutathione peroxidase 4 (GPX4), indicating the crucial involvement of the SLC7A11-GSH-GPX4 antioxidant axis in the NeuroD4-induced reprogramming process.

## Results

### NeuroD4 transforms human glioblastoma cells into neuron-like cells

We initially investigated the role of NeuroD4 in inducing neuronal reprogramming of human glioblastoma cells. U251 and KNS89 cells were cultured and infected with NeuroD4-expressing lentivirus. The infection efficiency of the GFP+NeuroD4 group was estimated to be more than 91% in U251 cells and 89% in KNS89 cells, based on the co-expression of GFP and nuclear marker DAPI. No significant difference was observed between the GFP+NeuroD4 and control GFP groups (Supplementary Fig. S1A–D). RNA sequencing confirmed the overexpression of NeuroD4 mRNA, and western blot analysis confirmed the expression of the Flag-NeuroD4 protein in U251, KNS89, and U87 cells infected with the GFP+NeuroD4 virus at 5 days post infection (dpi) (Supplementary Fig. S1E–G).

**Fig. 1:**
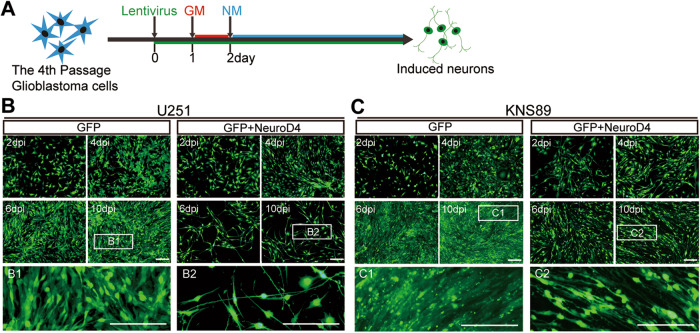
Morphological changes of human glioblastoma cells induced by NeuroD4. **A** Schematic representation of the cell reprogramming process. **B**, **C** Rapid morphological changes of U251 and KNS89 cells were observed upon ectopic expression of NeuroD4. The control group was infected with GFP-expressing virus. Higher magnification views of the boxed regions are also depicted. Dpi: days after infection; GM: medium for glioblastoma cells; NM: medium for neuronal induction. Scale bar: 200 µm.

As depicted in Fig. 1A, one day after infection with GFP and GFP+NeuroD4 lentivirus, the glioblastoma cell medium was replaced. The following day, neuron induction medium was used for further cell culture [8, 14]. Time-course morphological analysis demonstrated that NeuroD4 overexpression induced U251 and KNS89 cells to undergo morphological changes as early as 4 dpi (Fig. 1B, C). The cells lost their spindle or polygonal morphology and acquired a neuronal appearance with long neurites. By 6 and 10 dpi, they exhibited a more complex morphology with multiple neuron-like processes. In contrast, the GFP group did not show significant changes in cell shape (Fig. 1B, C).

**Fig. 2:**
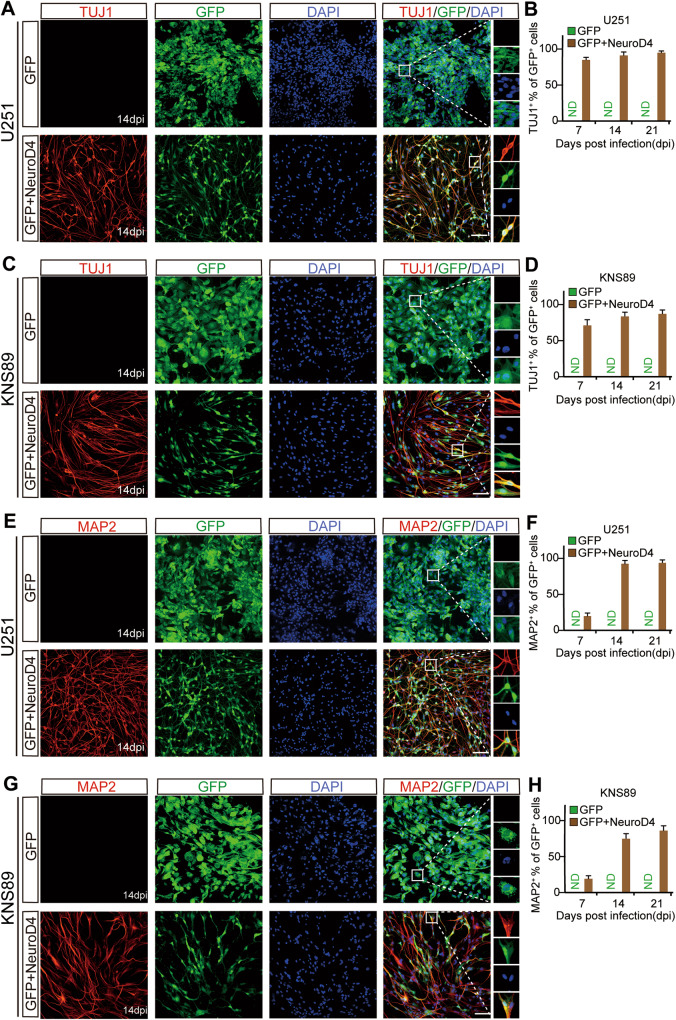
Time-course analysis of NeuroD4-mediated neuronal reprogramming of human glioblastoma cells. **A**, **C**, **E**, **G** Immunostaining of neuronal markers TUJ1 and MAP2 in NeuroD4 transformed U251 and KNS89 cells at 14 dpi. **B**, **D**, **F**, **H** Quantifying neuronal marker expression in NeuroD4-infected U251 and KNS89 cells during the indicated course (9 random fields from triplicate samples were captured for quantification). Dpi: days post infection. ND not detected. Scale: 100 µm.

The presence of TUJ1 and MAP2 in NeuroD4-transduced cells indicated the successful cell reprogramming action of NeuroD4 [20]. However, neither the TUJ1 nor MAP2 signal was detected in the GFP group (Fig. 2A, C, E, G). A time-course analysis of TUJ1 and MAP2 expression revealed their presence in GFP+NeuroD4 cells at 7 dpi, and their levels increased by 14 and 21 dpi (Fig. 2B, D, F, H). At 14 dpi in the GFP+NeuroD4 group, 92.8% of infected U251 cells and 84.8% of infected KNS89 cells were reprogrammed into TUJ1+ cells, while 94.1% of infected U251 cells and 75.8% of infected KNS89 cells were reprogrammed into MAP2+ neuron-like cells (Fig. 2B, D, F, H). At 21 dpi, the TUJ1 and MAP2 positive rates of U251 and KNS89 cells slightly increased (Fig. 2B, D, F, H). The high positive rates of TUJ1 and MAP2 in GFP+ cells of the GFP+NeuroD4 group lead us to conclude that NeuroD4 overexpression efficiently reprograms specific glioblastoma cell types into neuron-like cells.

### NeuroD4-induced neuronal reprogramming results in cell cycle exit and suppresses glioblastoma cells growth

Neurons are typically considered postmitotic cells that have undergone terminal differentiation and lack the ability to proliferate [21]. Therefore, we investigated whether NeuroD4-mediated neuronal reprogramming could inhibit the aggressive proliferation of glioblastoma cells. We assessed the proliferation of reprogrammed glioblastoma cells using the Ki67 protein and EdU labeling. Ki67 protein is highly expressed in cycling cells and is used to assess the growth of reprogrammed glioblastoma cells [22]. EdU (5-ethynyl-2’-deoxyuridine) is incorporated into newly synthesized DNA during cell proliferation, enabling sensitive and quantitative detection of proliferating cells [23].

**Fig. 3:**
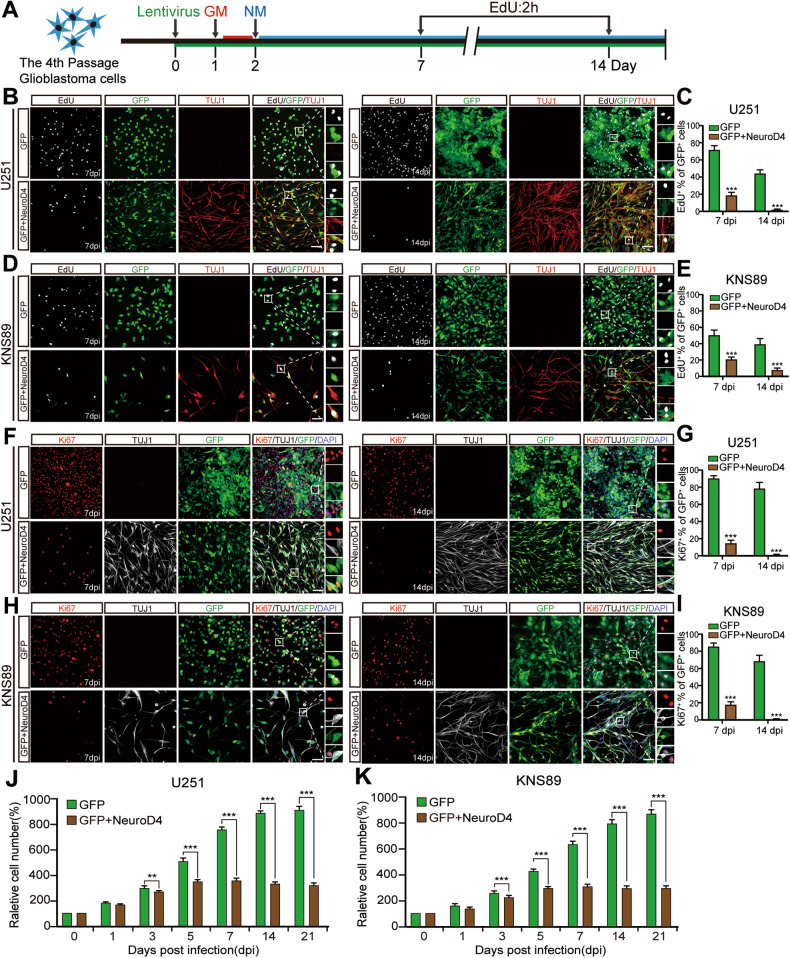
NeuroD4-induced neuronal reprogramming suppresses the proliferative marker EdU and Ki67 in glioblastoma cells. **A** The experimental design for labeling EdU in glioblastoma cells. **B**–**E** EdU detection of U251 and KNS89 cells at 14 dpi and quantitative analysis of EdU+ (9 random fields from triplicate samples were captured for quantification). **F**–**I** Immunocytochemical analysis of the Ki67 marker in U251 and KNS89 cells at 14 dpi and quantitative analysis of Ki67+ (9 random fields from triplicate samples were captured for quantification). **J**, **K** Time-course cell counting at 0, 1, 3, 5, 7, 14, and 21 dpi. The data are presented as mean ± SD. ***P < 0.001. Dpi: days post infection; GM glioblastoma cell medium, NM neuronal induction medium. Scale: 100 µm.

At 7 and 14 dpi, we labeled proliferating cells with EdU (10 µM) for a 2-hour incubation followed by a click reaction (Fig. 3A). We also performed immunocytochemical staining for the Ki67 protein at 14 dpi. At 7 dpi, compared to the GFP group, glioblastoma cells with forced expression of GFP+NeuroD4 showed a significant decrease in the number of EdU+ and Ki67+ cells (Fig. 3B–I), suggesting that NeuroD4-mediated neuronal reprogramming may induce cell cycle exit in U251 and KNS89 cells. At 14 dpi, the proportion of EdU+ cells decreased from 38.6% to 2.1% in infected U251 cells and from 37.4% to 1.5% in infected KNS89 cells (Fig. 3F–I). Additionally, the Ki67 protein was nearly absent in GFP+NeuroD4-infected cells (Fig. 3F–I). Although a small number of infected cells expressing TUJ1 or MAP2 did not exhibit neuron-like morphology and were not considered successfully reprogrammed, their proliferation was still inhibited, possibly due to dying or being in the early phase of reprogramming [24].

**Fig. 4:**
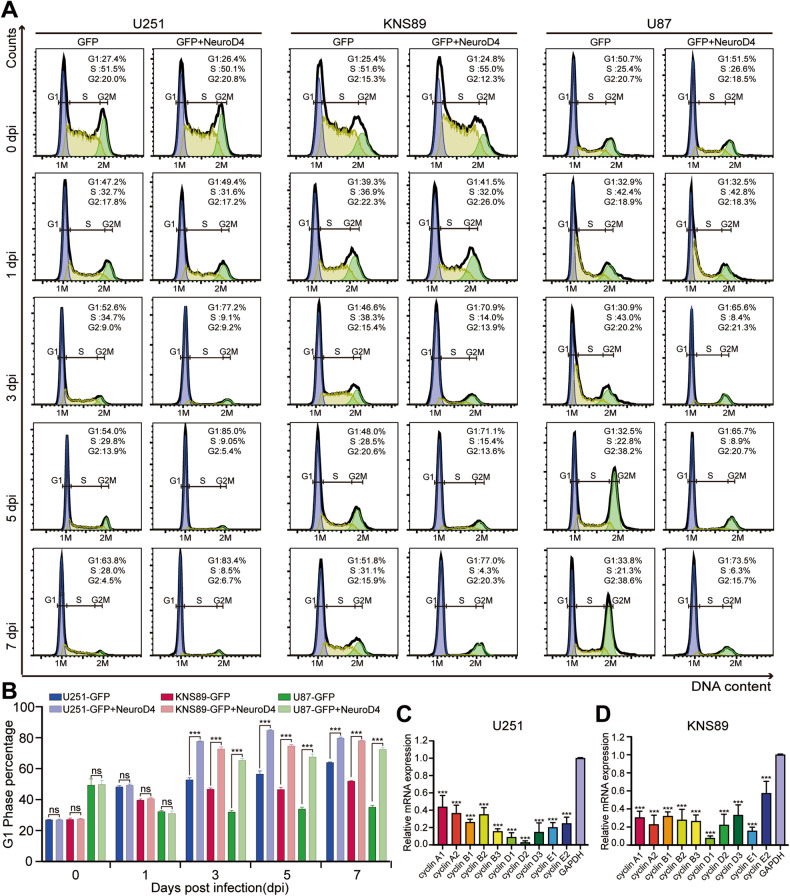
Overexpression of NeuroD4 resulted in cell cycle exit. **A** Time-course flow cytometric analysis of cell cycle displaying the cellular DNA content of NeuroD4 overexpressed cells in U251, KNS89, and U87 cells. **B** Quantitative analysis of DNA content (*n* = 3). **C**, **D** The expression of various cell cycle-related mRNA in U251 and KNS89 cells was detected by qRT-PCR. The data are presented as mean ± SD. **P < 0.01, ***P < 0.001 by student’s *t*-test and One-way analysis of variance (ANOVA). Dpi days post infection. NS no significance.

A time-course cell counting analysis revealed that the control group infected with GFP-expressing virus continued to exhibit aggressive growth, while the growth of glioblastoma cells infected with the GFP+NeuroD4 virus reached a plateau at 5 dpi, showing no significant development thereafter (Fig. 3J, K). To confirm that the cell cycle of GFP+NeuroD4 virus-infected glioblastoma cells was arrested in the early stages of reprogramming, we performed a time-course flow cytometry analysis by labeling the DNA of glioblastoma cells with PI dye. We also used qRT-PCR to detect the mRNA expression levels of cell cycle proteins at 5 dpi. In U251, KNS89, and U87 cells, compared to the GFP group, the proportion of cells in the stable G1 phase significantly increased at 3, 5, and 7 dpi, while the proportion of cells in S phase and G2/M phase decreased markedly (Fig. 4A, B). Furthermore, the expression of various cell cycle-related mRNA in U251 and KNS89 cells significantly decreased at 5 dpi (Fig. 4C, D). These findings indicate that NeuroD4-induced neuronal reprogramming can inhibit the exponential growth of glioblastoma cells, leading to a significant reduction in multiple cyclins, and causing reprogrammed glioblastoma cells to exit the cell cycle as early as 3 dpi.

### NeuroD4 inhibits the human-derived glioblastoma xenograft development in vivo through reprogramming

**Fig. 5:**
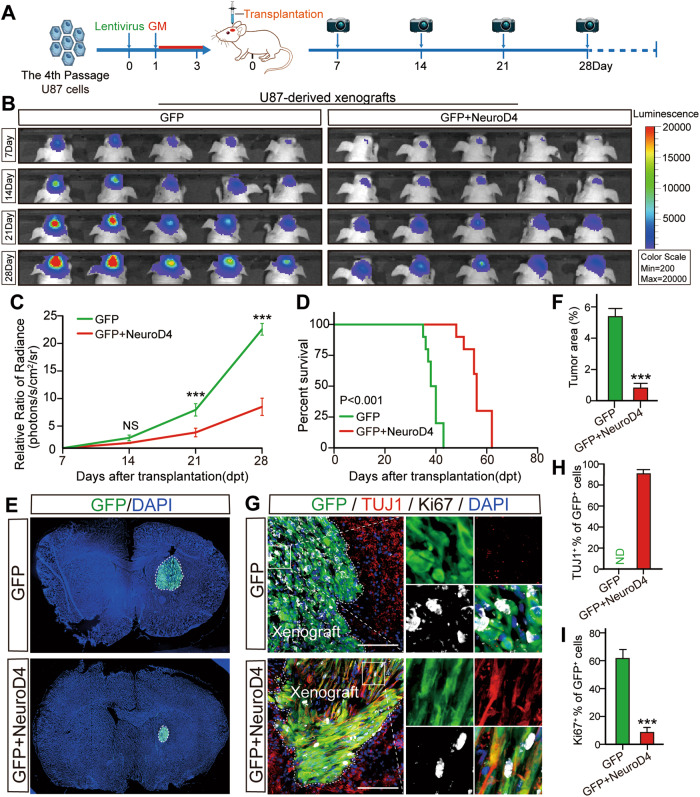
The xenografts derived from NeuroD4 overexpressing U87 cells shrink significantly. **A** The experimental protocol for orthotopic cell transplantation. **B**, **C** In vivo bioluminescent images and the quantification of U87-derived xenografts (*n* = 5). **D** Post-imaging Kaplan-Meier survival analysis of transplanted mice (*n* = 10, *P* < 0.001 using log-rank test). **E**, **F** Immunohisto fluorescence analysis of tumor size in mice implanted with GFP and GFP+NeuroD4 virus infected U87 cells 28 days after transplantation (*n* = 6). Tumor mass (outlined by dashed lines) was quantified based on the area occupying the ipsilateral brain. **G**–**I** Ki67 and TUJ1 markers of NeuroD4 overexpressing U87 cells in xenografts 28 days after implantation. Forty-five random fields from a series of every tenth coronal brain section of six nude mice were collected for quantification in Immunohisto fluorescence analysis. The data are presented as mean ± SD. ***P < 0.001 by student’s *t*-test and One-way analysis of variance (ANOVA). NS: no significance. ND not detected. GM glioblastoma cell medium. Scale: 100 µm.

To assess the impact of NeuroD4 overexpression on tumor growth in vivo, we selected U87 cells carrying a luciferase reporter gene, known for forming brain masses (Fig. 5A). In vitro, we infected cultured human U87 cells with GFP+NeuroD4 or control GFP-expressing lentivirus. Three days later, we transplanted these cells into the striatum of nude mice and monitored tumor growth using the PerkinElmer IVIS Lumina X5 in vivo imaging system every seven days. Survival time was recorded daily. The results revealed a significant inhibition of GFP+NeuroD4 tumor growth in the brain (*n* = 5, *p* < 0.001, Fig. 5B, C), leading to a significant prolongation of survival time in the nude mice up to 60 days (*n* = 10, *p* < 0.001, Fig. 5D).

At 28 days post-transplantation, we obtained frozen brain sections from nude mice and observed that the size of GFP+NeuroD4 tumors in the ipsilateral brain was smaller compared to tumors caused by GFP-only transduced glioblastoma cells (*n* = 6, Fig. 5E, F). Additionally, we performed double staining of Ki67 with TUJ1 to evaluate reprogramming and aggressive proliferation of U87 cells infected with the GFP+NeuroD4 virus in vivo. We found that 90.9% of the GFP+NeuroD4 group expressed the early neuronal marker TUJ1, whereas no TUJ1+ cells were observed in the brains of the control GFP group (Fig. 5G–I). Approximately 60% of control GFP-expressing U87 cells stained positive for Ki67, while less than 10% of NeuroD4-expressing U87 cells were Ki67+ (Fig. 5G–I). Furthermore, we observed 6.3% of GFAP+ Ki67+ cells in the GFP+NeuroD4 group, but we did not detect SOX2, SOX10, and OLIG2 (Supplementary Fig. S2A–F) in the xenograft. Low-power photographs of the entire brain are shown in Supplementary Fig. S2G.

These findings collectively demonstrate that the single transcription factor NeuroD4 can reprogram human glioblastoma cells into terminally differentiated neuron-like cells in the brains of nude mice, leading to the inhibition of aggressive glioblastoma proliferation.

### Ferrostatin-1 inhibits the NeuroD4-mediated reprogramming

**Fig. 6:**
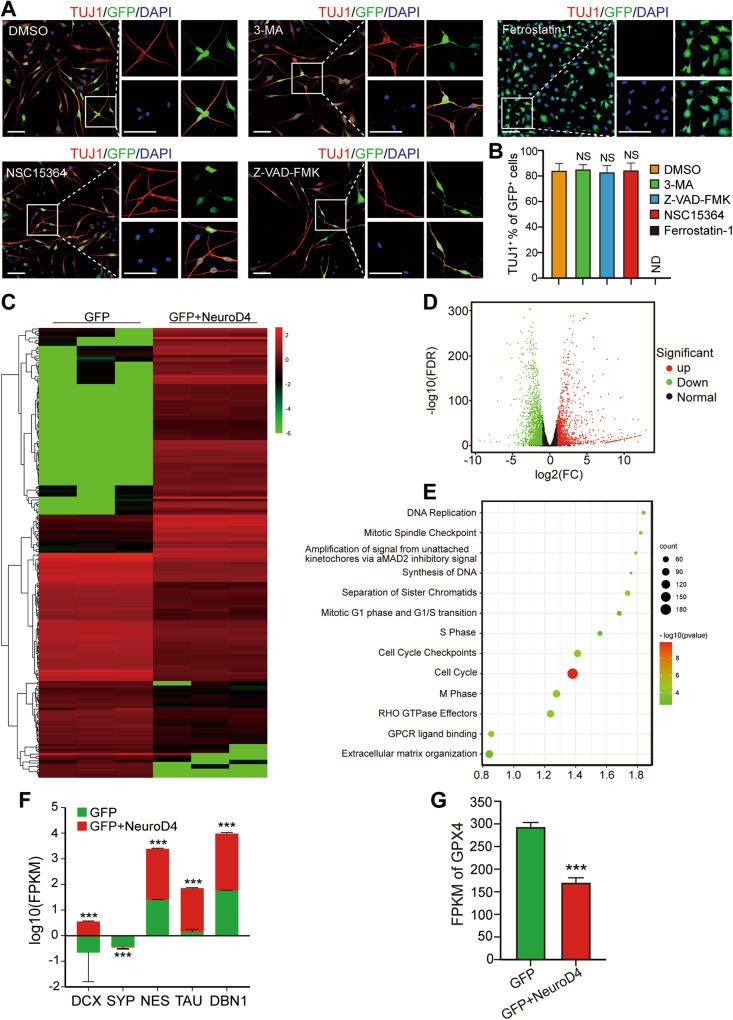
Screening of drugs for inhibiting cell death and analysis of RNA sequencing. **A**, **B** TUJ1 detection at 7 dpi revealed the reprogramming efficiency of GFP+NeuroD4 lentivirus infected U251 cells treated with four cell death inhibitors and quantitative analysis (9 random fields from triplicate samples were captured for quantification). **C** A heatmap shows the expression of 200 distinct genes in GFP and GFP+NeuroD4 U251 cells (*n* = 3). **D**, **E** The volcano map and pathway enrichment analysis of distinct expression genes between GFP and GFP+ NeuroD4 U251 cells. **F** The log10 (FPKM) value of neuronal markers in glioblastoma cell reprogramming (*n* = 3). **G** The FPKM of GPX4 between GFP and GFP+ NeuroD4 U251 cells (*n* = 3). The data are presented as mean ± SD. ***P < 0.001 by student’s *t*-test. Dpi days post infection. NS no significance. ND not detected. Scale: 100 µm.

To investigate the mechanism underlying NeuroD4-induced reprogramming and inhibition of cell proliferation, we employed inhibitors to block ferroptosis (ferrostatin-1, 10 µM), autophagy (3-MA, 100 µM), apoptosis (NSC 15364, 50 µM), and pyroptosis (Z-VAD-FMK, 20 µM) during the transition to C2 medium. In NeuroD4 virus-infected U251 cells, the ferrostatin-1 group showed minimal expression of TUJ1 seven days after infection. However, the other inhibitor groups, as well as the DMSO group, exhibited a TUJ1 positive rate of over 80%, with no significant difference (Fig. 6A, B). These results indicate that ferrostatin-1 can suppress NeuroD4-mediated neuronal reprogramming in glioblastoma. Additionally, at 5 dpi, we assessed the expression of BCL-2 and BAX proteins in all groups via western blot to determine if the inhibitors used here affect cell death. However, no significant differences were observed in BCL-2 and BAX protein expression between the DMSO group and other inhibitor groups (Supplementary Fig. S2H–J), suggesting that the inhibitors employed did not affect apoptosis.

To further elucidate the mechanism by which NeuroD4 overexpression affects glioblastoma cell reprogramming, we isolated total RNA from U251 cells infected with GFP and GFP+NeuroD4 viruses, five days after infection, for sequencing. A total of 3305 genes exhibited differential expression between the GFP and GFP+NeuroD4 groups, with 1515 genes upregulated and 1790 genes downregulated. We extracted the log10 (FPKM) values of the 3305 genes with significant differences (Fold Change > 2) and plotted the top 100 upregulated and 100 downregulated genes in a heatmap, revealing substantial changes in the cell transcriptome (Fig. 6C). Additionally, we created a volcano plot to visualize the changes in transcriptome between the GFP and GFP+NeuroD4 groups. Pathway enrichment analysis using Reactome (v78) identified critical genes and revealed enrichment in DNA replication, the mitotic spindle checkpoint, the cell cycle pathway, and high expression of neuronal development markers such as DCX, SYP, NES, TAU, and DBN1 (Fig. 6D–F). These findings align with the observation of glioblastoma cells undergoing transformation into neuron-like differentiated cells. Notably, the mRNA expression of GPX4, which provides protection against overoxidation and prevents cells from undergoing ferroptosis, exhibited a significant decrease in NeuroD4-overexpressing glioblastoma cells (Fig. 6G).

### Blocking the SLC7A11-GSH-GPX4 antioxidant axis promotes reprogramming caused by NeuroD4

**Fig. 7:**
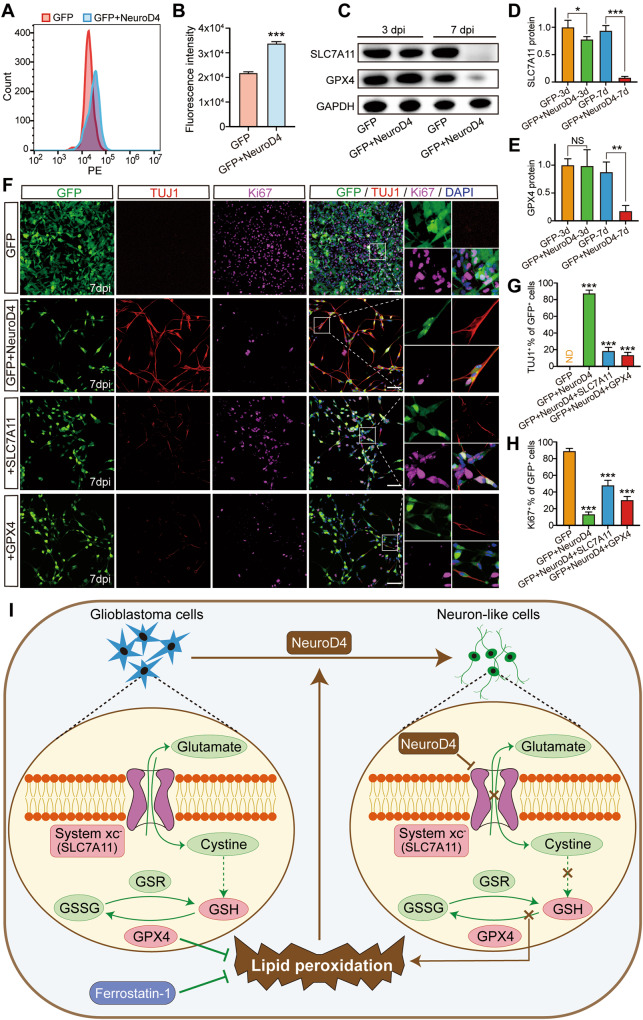
SLC7A11-GSH-GPX4 antioxidant axis is a key in NeuroD4-mediated neuronal reprogramming. **A**, **B** The fluorescence intensity of Reactive oxygen species (ROS) was detected by flow cytometry at 5 dpi. **C**–**E** Western blot analysis of SLC7A11 and GPX4 protein in U251 cells infected with GFP and GFP+NeuroD4 virus at 3 and 7 dpi. (*n* = 3). **F**–**H** Ki67 and TUJ1 staining revealed the reprogramming efficiency of particular lentiviruses-infected U251 cells at 14 dpi (9 random fields from triplicate samples were captured for quantification). **I** Pathways involved in NeuroD4-induced reprogramming. GSR glutathione-disulfide reductase. GSSG glutathione oxidized. The data are presented as mean ± SD. *P < 0.01, **P < 0.005, ***P < 0.001 by student’s *t*-test. Dpi days post infection. NS no significance. ND not detected. Scale: 100 µm.

Glutathione peroxidase 4 (GPX4), also known as phospholipid hydroperoxide glutathione peroxidase PHGPx, is an intracellular antioxidant enzyme that directly reduces peroxidized phospholipids in the cell membrane [25]. The SLC7A11-glutathione-GPX4 axis, which is part of the antioxidant system, plays a crucial role in preventing lipid peroxidation-mediated ferroptosis [26]. In our experiments, we utilized the U251 cell line for further investigation. At 5 dpi, we labeled the cells with dihydroethidium and measured the fluorescence intensity of reactive oxygen species (ROS) using flow cytometry. Compared to the GFP group, the GFP+NeuroD4 group showed a significant rightward shift in the peak of the PE channel and an increase in fluorescence intensity (Fig. 7A, B), indicating the accumulation of ROS in glioblastoma cells following NeuroD4 overexpression.

As shown in Fig. 7C–E, the protein expression of SLC7A11 and GPX4 decreased significantly in the GFP+NeuroD4 group at 7 dpi. To investigate whether SLC7A11 and GPX4 inhibit NeuroD4-mediated neuronal reprogramming in glioblastoma cells, we co-overexpressed SLC7A11 and GPX4 along with NeuroD4 (Supplementary Fig. S1H–K). At 7 dpi, the TUJ1+ rate in the GFP+NeuroD4 + SLC7A11 group and the GFP+NeuroD4 + GPX4 group decreased from 87.6% to 18.4% and 13.6%, respectively, while the Ki67+ rate increased from 13.3% to 48.1% and 30.3%, respectively, compared to the GFP+NeuroD4 group (Fig. 7F–H). These findings suggest that overexpression of SLC7A11 and GPX4 inhibits NeuroD4-mediated neuronal reprogramming in glioblastoma cells. Based on these results, we can conclude that induced ferroptosis generally promotes NeuroD4-mediated reprogramming through the SLC7A11-GSH-GPX4 antioxidant axis (Fig. 7I).

## Discussion

The use of reprogramming technology to transform tumor cells into terminally differentiated cells presents a promising strategy for tumor therapy [13, 19, 20]. In our study, we specifically employed NeuroD4 to reprogram a glioblastoma cell line, resulting in the conversion of these cells into neuron-like cells. In vivo experiments demonstrated that the reprogrammed glioblastoma cells underwent terminal differentiation, leading to the inhibition of proliferation and exit from the cell cycle. Additionally, NeuroD4 virus-infected xenografts exhibited smaller tumor sizes compared to the control GFP group, and the survival time of tumor-bearing mice in the GFP+NeuroD4 group was prolonged. Mechanistically, NeuroD4 overexpression significantly reduced the levels of SLC7A11 and Glutathione peroxidase 4 (GPX4), suggesting their involvement in the reprogramming process. Furthermore, the ferroptosis inhibitor ferrostatin-1 was found to effectively block the neuron reprogramming process mediated by NeuroD4 in glioblastoma.

Neurog2 and ASCL1, both basic helix loop helix (bHLH) transcription factors, have been shown to reprogram glioblastoma cells into neuron-like cells [19, 20]. NeuroD4, as a downstream transcription factor of Neurog2 and ASCL1, has the capacity to induce neuronal reprogramming of various cell types, including fibroblasts and human astrocytes [15]. NeuroD4 has also demonstrated its ability to enhance morphological and functional recovery following spinal cord injury, highlighting its role in neuroregeneration [27]. In our study, NeuroD4 successfully reprogrammed U251 and KNS89 glioblastoma cell lines into a neural differentiation state, achieving an efficiency of over 90%, which is comparable to the reprogramming efficiency achieved by overexpressing ASCL1 or knocking down PTBP1 [19, 28].

Uncontrolled cell proliferation is a hallmark of glioblastoma pathogenesis. In our study, we observed a significant inhibition of proliferation in the successfully reprogrammed malignant glioblastoma cells, aligning with our objective of delaying tumor progression through NeuroD4 overexpression. The cell cycle analysis revealed a G1 phase block in NeuroD4 virus-infected glioblastoma cells, accompanied by a substantial reduction in the mRNA expression of multiple cyclins. RNA-seq results indicated the involvement of DNA replication, mitotic spindle checkpoint, and cell cycle pathways, further supporting the effective blockade of glioblastoma proliferation by NeuroD4-mediated neuronal reprogramming.

Recently, several signaling pathways have emerged as important regulators of neuronal reprogramming. One such pathway is the NOTCH1 signaling pathway, which has been identified as a key barrier to direct neuronal reprogramming in astrocytes [29]. In our study, we uncovered the crucial role of ferroptosis in NeuroD4-mediated neuronal reprogramming of glioblastoma. Ferroptosis is a form of iron-dependent cell death that is driven by reactive oxygen species (ROS) [30]. The role of ferroptosis in tumorigenesis is still not fully understood [31]. However, aberrant ferroptosis has been observed in various cancer types, including hepatocellular carcinoma, colorectal cancer, gastric cancer, ovarian cancer, breast cancer, and lung cancer [32–36]. The anti-tumor effects of ferroptosis have also been demonstrated in the treatment of glioma using drugs like temozolomide [37, 38]. Additionally, ferroptosis has been shown to inhibit tumor development in response to treatments with cisplatin, sorafenib, and other anti-tumor drugs in different malignancies [39–42]. Recent studies have implicated ferroptosis in cell death mechanisms in malignant brain tumors [43]. Taken together, these findings suggest that the ferroptosis pathway plays a critical role in the clinical treatment of malignant tumors, including glioblastoma.

In our research, we observed an increase in ROS levels in GFP+NeuroD4 virus-infected cells, which also promoted cell ferroptosis (Fig. 7A, B). ROS has been shown to regulate stem cell fate, and elevated ROS levels resulting from oxidative stress are crucial mediators of many cellular and developmental processes [44, 45]. Previous studies have confirmed the role of ROS in cell differentiation. For example, ROS promotes differentiation of acute promyelocytic leukemia cells [46], and ROS is involved in isoliquiritigenin-induced monocytic differentiation in human acute promyelocytic leukemia HL-60 cells [47]. ROS primarily act through various targets, including kinases and transcription factors, and exhibit diverse roles in different stages of cardiac differentiation. Endogenous ROS production in the early differentiation state suppresses endoderm differentiation through transient FOXC1 expression [48].

However, the intrinsic mechanism linking ferroptosis and reprogramming has not been fully elucidated. In our study, we found that the positive expression of SLC7A11 and GPX4, which reduce phospholipid hydroperoxide and prevent ferroptosis, was decreased in U251 glioblastoma cells (Fig. 7C–E). During the reprogramming process, NeuroD4 inhibits the function of the cystine/glutamate antiporter system xc− which includes the key subunit SLC7A11 [26]. This inhibition hinders glutamate transport into cells, resulting in decreased synthesis of glutathione (GSH), leading to the depletion of GPX4 and subsequent ferroptosis characterized by lipid peroxidation. Ultimately, this promotes the reprogramming of glioblastoma into neuron-like cells (Fig. 7H). Our findings suggest that induced ferroptosis promotes NeuroD4-mediated reprogramming through the SLC7A11-GSH-GPX4 antioxidant axis. This discovery paves the way for future mechanistic studies on reprogramming and ferroptosis.

In summary, our study provides insights into the molecular basis of NeuroD4-mediated reprogramming of glioblastoma into neuron-like cells. This breakthrough offers a new approach for anti-glioblastoma therapies. However, several challenges need to be addressed before the clinical application of reprogramming technology in glioblastoma treatment, including improving the efficiency of conversion and enhancing the specificity of viral transduction for glioblastoma targeting.

## Materials and methods

### Animals and ethics consideration

The immunodeficient nude mice used in our study were all provided by the Shanghai Charles River Experimental Animal Limited Liability Company (Shanghai, China). These animals were housed in a controlled environment with free access to food and water. Following the National Institutes of Health’s animal care and use guidelines, the Animal Ethics Committee of Wenzhou Medical University approved all experimental procedures and protocols strictly.

### Model and imaging system for a brain tumor

Nude male mice were sequentially numbered and randomly divided into two groups (*n* = 16). Three days later, the infected U87 cells (5 × 10^5^ cells in 4 µL) were stereotactically injected into the striatum of nude mice (*n* = 16) according to the injection coordinates as follows: anterior/posterior, +1.0 mm; medial/lateral, +2.0 mm; and dorsal/ventral from the skull, −3.0 mm.

We used PerkinElmer IVIS Lumina X5 (USA) in vivo imaging system to evaluate the growth of tumor mass every 7 days (*n* = 5) and recorded the natural death date for survival analysis (*n* = 10). Moreover, IHF samples were taken 28 days after transplantation. Tumor sizes were quantified from 10 representative 10-μm-thick serial coronal brain sections. All measurements and observations were performed by a blinded researcher.

### Cell culture

HEK-293T cells and human glioblastoma cells (U251, U87, and KNS89) were grown in Dulbecco’s Modified Eagle Medium (DMEM) (11995040, Gibco, USA) containing 10% fetal bovine serum (FBS) (10099141, Gibco, USA) and 1% penicillin/streptomycin (15140122, Gibco, USA). U251, U87, and KNS89 cells were the fourth passage cells when infected with GFP and GFP+NeuroD4 viruses. After coating with matrigel (354234, BD, USA), Confocal laser scanning microscopy-compatible 24-well plates with ultra-thin bottoms (P24-1.5H-N, Cellvis, USA) were used to cultivate glioblastoma cells. Two days after infection with the virus packed in HEK-293T, neuronal induction medium consists of DMEM, F12, and neurobasal (2:2:1, 11995040, 11765054, 21103049, Gibco, USA), N2 (17502001, Gibco, USA), B27 (17504044, Gibco, USA), forskolin (10 µM, S2449, Selleck, USA), and dorsomorphin (1 µM, S7840, Selleck, USA) was used to cultivate these cells until the end of reprogramming. We utilized ferrostatin-1 (10 µM, HY-100579, MCE, USA) to inhibit cell ferroptosis, 3-MA to inhibit autophagy (100 µM, HY-19312, MCE, USA), NSC 15364 (50 µM, HY-108937, MCE, USA) to inhibit apoptosis and Z-VAD-FMK (20 µM, HY-16658B, MCE, USA) from inhibiting pyroptosis. To induce and block ferroptosis, erastin (10 µM, HY-15763) and ferrostatin-1 (10 µM, HY-100579) acquired from MCE (USA) were employed. The induction medium was changed every other day during the reprogramming process.

### Plasmid assembly and lentiviral packaging

FLAG tagged NeuroD4, SLC7A11, GPX4 plasmid started by CMV promoter and control vector were provided by Youze Biology (China). The NeuroD4 lentivirus encodes a human variant and GFP encoded by NeuroD4 lentivirus was used to visualize virus-infected cells. The lentiviral vectors and packaging plasmids (pMDL, VSV-G, and pRSV) were then transfected into HEK-293T cells, and the media were replaced after 16 h. After 24 and 48 h, virus media was collected, filtered, and supplemented with 10 ng/mL polybrene (TR-1003-G, Milipore, USA), and glioblastoma cell lines were infected. Unaware operators used these viruses to interfere with the overexpression of NeuroD4-induced reprogramming, followed by ICF staining.

### Western blot analysis

We collected U251 cells and tissue samples and used RIPA lysis buffer (89900, Thermo Fisher Scientific, USA) to extract total proteins. Then measured them using a bicinchoninic acid (BCA) Protein Assay Kit (23227, Thermo Fisher Scientific, USA). Sodium dodecyl sulfate-polyacrylamide gel electrophoresis separated the protein samples (*n* = 3), followed by their transfer onto polyvinylidene fluoride membranes. After 2 h of blocking in 5% non-fat milk, membranes were probed overnight at 4 °C with primary antibodies against FLAG (66008-4-Ig, proteintech, China, 1:5000), BAX (50599-2-Ig, proteintech, China, 1:2000), BCL-2 (68103-1-Ig, proteintech, China, 1:2000), GPX4 (ab125066 abcam, USA, 1:1000); SLC7A11 (DF12509, Affinity, USA, 1:1000), GAPDH (AF7021, Affinity, USA, 1:1000), and 2 h of secondary antibody (PR30011/PR30012, proteintech, China, 1:5000) incubation. Proteins were visualized by enhanced chemiluminescence (MA0186, Meilunbio, China) on a ChemiDoc camera (Bio-Rad), and protein expression levels were quantified using the ImageJ software (v1.8.0). Full and uncropped western blots are presented in Supplemental File.

### Real-time quantitative PCR (qRT-PCR)

Total RNA was extracted by TRIzol reagent (15596018, Thermo Fisher Scientific, USA). Then 2 μg RNA per sample (*n* = 3) was converted to cDNA using Revert Aid First Strand cDNA Synthesis Kit (K1622, Thermo Fisher Scientific, USA). Primer-BLAST was used to design the primers for all target genes manufactured by Sangon Biotech (China). The primers list used in this study is reported in Supplementary Table S1. For real-time gene expression quantification, Iraq Universal SYBR Green supermix (1725124, BIO-RAD, USA) was used. The expression levels of cyclins were normalized to GAPDH and computed by the 2^–∆∆Ct^ method.

### Immunofluorescence (IF)

Brain tissues were fixed by left ventricle perfusion for immunohistochemistry with 4% paraformaldehyde in PBS. Brain tissue was then collected for dehydration, fixation, and frozen sectioning. Glioblastoma cells and brain slices were fixed for 20 min in 4% paraformaldehyde at room temperature, then incubated for 1 h in PBST (0.2% Triton X-100 in PBS) containing 5% bovine serum albumin (BSA) (ST025, Beyotime, China) solution to inhibit non-specific staining. After that, samples were incubated at 4 °C overnight with primary antibodies (TUJ1 (ab78078), MAP2 (ab96378), Ki67 (ab15580) and GFAP (ab 68428) from Abcam, USA, SOX2 (11064-1-AP), SOX10 (10422-1-AP) and OLIG2 (13999-1-AP) from Proteintech, China) and then at 37 °C for 1 h with secondary antibodies (ab150080/ab150116, Abcam, USA, 1:1000). The nuclei were counterstained with DAPI (ab285390, Abcam, USA) for 15 min. The blinded researcher used confocal laser scanning microscopy (OLYMPUS, JPN) and Image J software (v1.8.0) to scan and analyze ICF or IHF staining. Nine random fields from triplicate cell samples or 45 random fields from a series of every tenth coronal brain section of six nude mice were collected for quantification.

### Cell proliferation assays

EdU (5-ethynyl-2’-deoxyuridine) incorporation assay was performed to determine cell proliferation. Cell proliferation was detected using BeyoClickTM EdU Cell Proliferation Kit with Alexa Fluor 647 (C0081S, Beyotime, China). EdU was administered to the cell culture medium (10 μM, 2-h incubation) in advance and then tagged with Alexa Fluor 647 through a click reaction.

Time-course cell counting was used to assess glioblastoma cell growth. Briefly, glioblastoma cells were seeded in a 24-well plate (2 × 10^4^ cells/well). The cell numbers were counted by a blinded researcher at 0, 1, 3, 5, 7, 14, and 21 days after infection with lentivirus.

### Flow cytometry

Glioblastoma cells were seeded in six-well plates (1 × 10^5^ cells/well) to 5 dpi. We used PI dye (CA1510, Solarbio, China) for the cell cycle analysis of U251, U87, and KNS89 cells, and dihydroethidium (S0063, Beyotime, China) for the ROS analysis of U251 cells 30 min before flow cytometry detection. Then, the fluorescence intensity was recorded by Cytoflex LX (Beckman Coulter, USA), and the data were analyzed using FlowJo software (v10.4.0).

### RNA sequencing (RNA-seq)

The raw data from Illumina HiSeq sequencing was filtered and compared to the reference sequence, which is the foundation of quantitative analysis of known and novel genes. Differentially expressed genes (Fold Change > 2, FDR < 0.01 in GFP vs. GFP+NeuroD4) between samples (*n* = 3) were sorted according to FDR and then excavated by STRING (v11.5). Sequencing data have been submitted to the national center for biotechnology information (NCBI) Gene Expression Omnibus under the accession number GSE210717. Furthermore, the FPKM value file is provided in the supplementary materials (Supplementary Table S2).

### Statistical analysis

All experimental data are expressed as standard deviation (SD). The statistical analysis was conducted using Graph Pad Prism software (v9.0.1). The normal distribution and difference between the two groups was determined by the student’s *t*-test. One-way analysis of variance (ANOVA) was used to compare three or more groups. The log-rank (Mantel-Cox) test was performed to compare Kaplan-Meier survival curves. *P* < 0.05 was considered statistically significant (*P < 0.01, **P < 0.005, ***P < 0.001).

### Supplementary information

Supplementary table legends

Supplementary figure legends

Supplementary Figure S1

Supplementary Figure S2

Supplementary Table S1

Supplementary Table S2

Original Western Blot Images

## Data Availability

The datasets used during the study are available from the corresponding author on request.
