# Supplementary material for: NeuroD4 converts glioblastoma cells into neuron-like cells through the SLC7A11-GSH-GPX4 antioxidant axis
Source: Cell Death Discov. 2023 Aug 15;9:297. doi: 10.1038/s41420-023-01595-8 (PMC10427652; doi:10.1038/s41420-023-01595-8)
Supplement: Supplementary file 1 — Supplementary table legends [file 41420_2023_1595_MOESM1_ESM.docx]

**Supplementary Tables**

**Supplementary Table S1**. Primer sequences used for qRT-PCR.

**Supplementary Table S2.** Differentially expressed genes (Fold Change > 2, FDR < 0.01 in GFP vs. GFP+NeuroD4) were listed in this table by mRNA-seq.
