# Supplementary material for: NeuroD4 converts glioblastoma cells into neuron-like cells through the SLC7A11-GSH-GPX4 antioxidant axis
Source: Cell Death Discov. 2023 Aug 15;9:297. doi: 10.1038/s41420-023-01595-8 (PMC10427652; doi:10.1038/s41420-023-01595-8)
Supplement: Supplementary file 2 — Supplementary figure legends [file 41420_2023_1595_MOESM2_ESM.docx]

**Supplementary figures**

**Supplementary Figure S1**. The infection efficiency of infected cells and the expression of NeuroD4, SLC7A11 and GPX4 mRNA and Flag protein. (**A**, **B**, **C**, and **D**) DAPI staining of infected U251 and KNS89 cells at 3 dpi and quantitative analysis (9 random fields from triplicate samples were captured for quantification). (**E**) The NeuroD4 FPKM value of RNA sequencing in U251 cells at 5 dpi. (**F** and **G**) Western blot analysis of FLAG protein in U251, KNS89 and U87 cells infected with GFP and GFP+NeuroD4 virus at 3 dpi (n = 3). (**H** and **I**) qRT-PCR for SLC7A11 and GPX4 mRNA in U251 cells infected with vector, SLC7A11 and GPX4 virus at 3 dpi (n = 3). (**J** and **K**) Western blot analysis of FLAG protein in U251 cells infected with vector, SLC7A11 and GPX4 virus at 3dpi (n = 3). The data are presented as mean ± SD. *** P < 0.001 by student's t-test. Dpi: days post infection. ND: not detected. Scale: 200 µm.

**Supplementary Figure S2.** There are about 5% GFAP+ cells of GFP+ cells in U87-derived xenograft and the inhibitors we used don’t affects apoptosis. (**A**-**F**) Double staining of Ki67 with GFAP, TUJ1, SOX2, SOX10 and OLIG2. (**G**) The low-power photographs of the whole brain double stained with immunofluorescence. (**H**-**J**) Western blot analysis of BCL-2 and BAX protein in U251 cells which infected with GFP+NeuroD4 virus and treated with four inhibitors at 3 dpi (n = 3). Dpi: days post infection. ND: not detected. Scale: 100 µm.
