# Supplementary figures and images for: NeuroD4 converts glioblastoma cells into neuron-like cells through the SLC7A11-GSH-GPX4 antioxidant axis
Source: Cell Death Discov. 2023 Aug 15;9:297. doi: 10.1038/s41420-023-01595-8 (PMC10427652; doi:10.1038/s41420-023-01595-8)

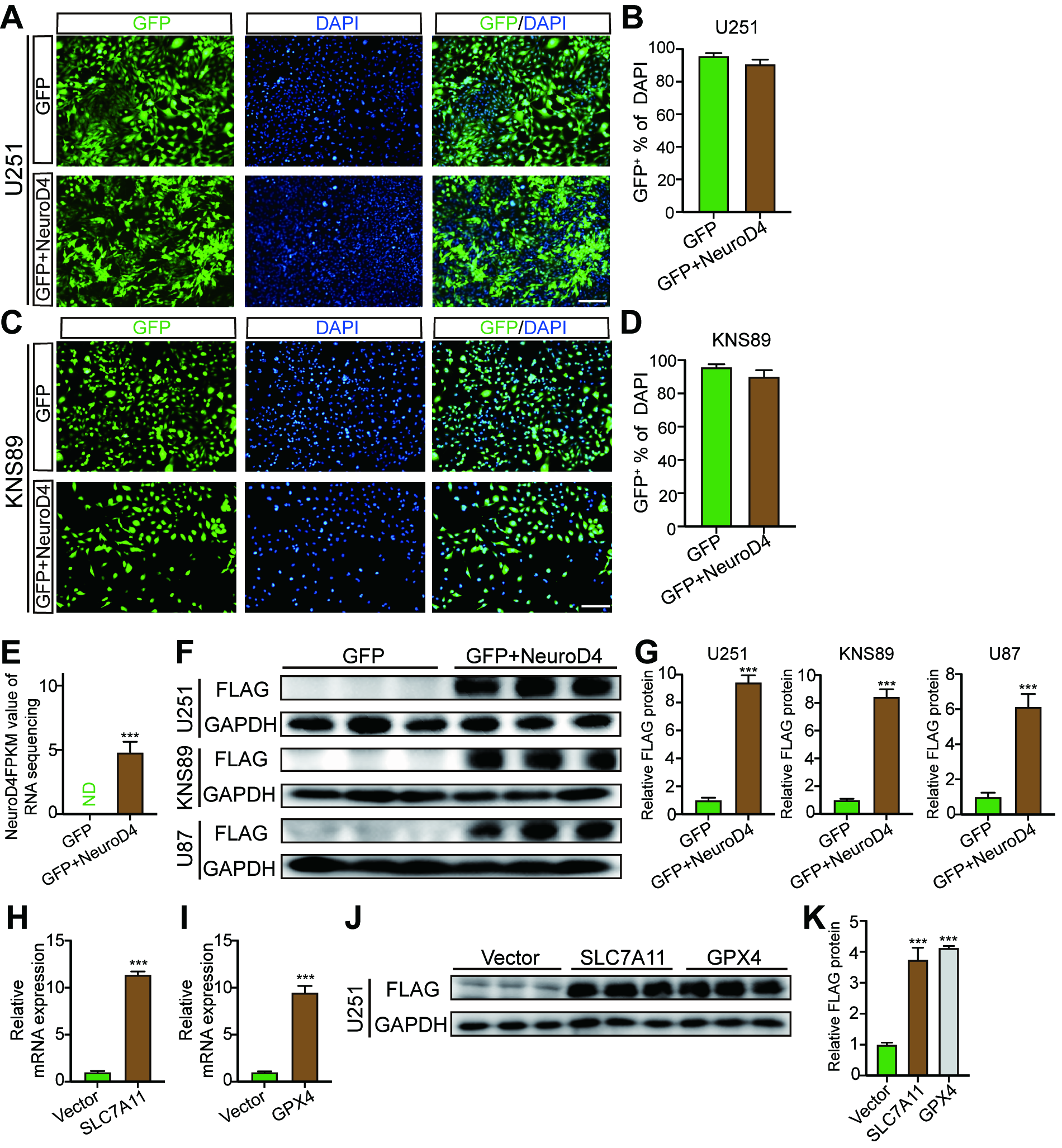

Supplement: Supplementary file 3 — Supplementary Figure S1 [file 41420_2023_1595_MOESM3_ESM.tif]

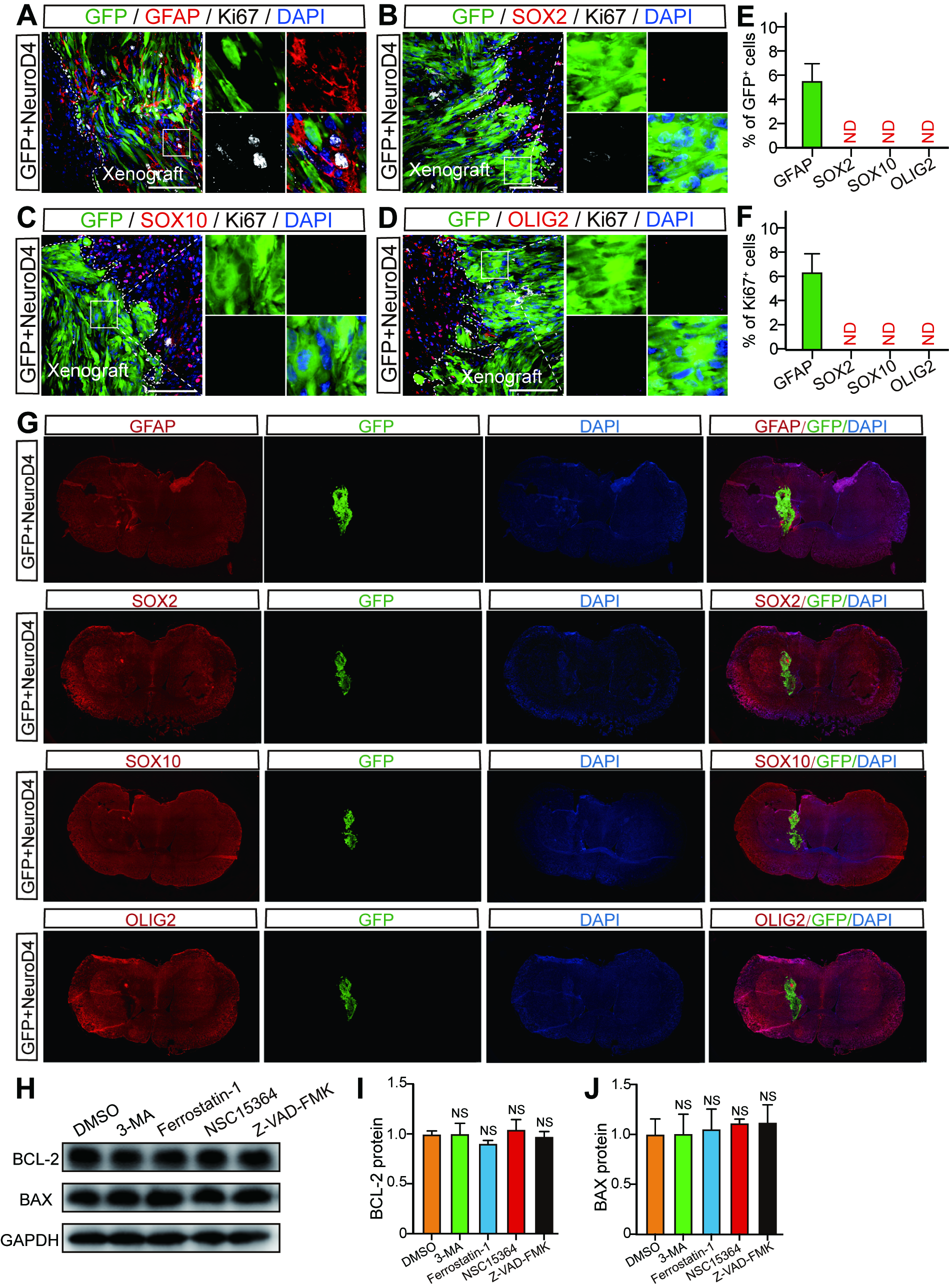

Supplement: Supplementary file 4 — Supplementary Figure S2 [file 41420_2023_1595_MOESM4_ESM.tif]

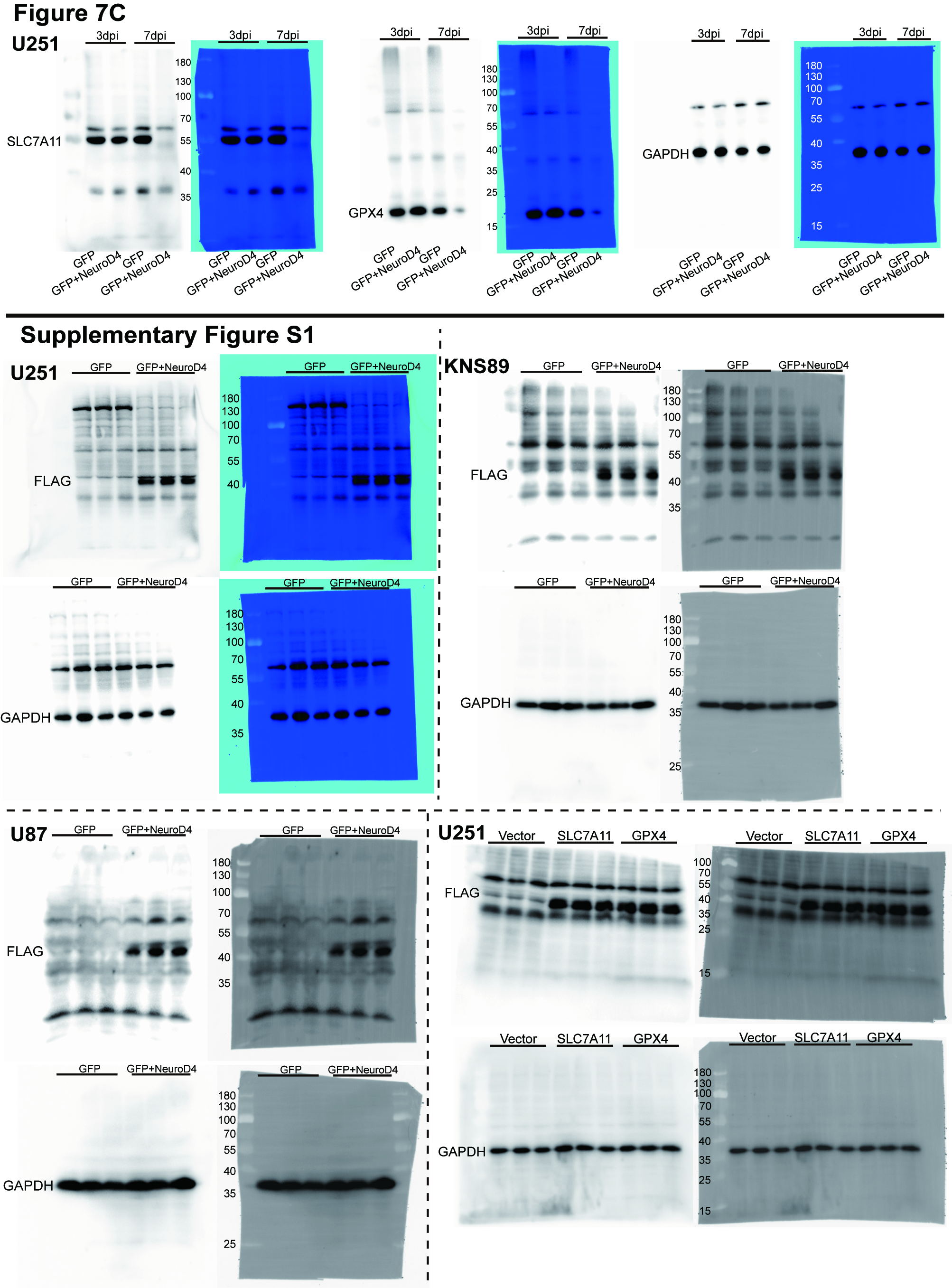

Supplement: Supplementary file 7 — Original Western Blot Images [file 41420_2023_1595_MOESM7_ESM.tif]
