# Supplementary material for: NeuroD4 converts glioblastoma cells into neuron-like cells through the SLC7A11-GSH-GPX4 antioxidant axis
Source: Cell Death Discov. 2023 Aug 15;9:297. doi: 10.1038/s41420-023-01595-8 (PMC10427652; doi:10.1038/s41420-023-01595-8)
Supplement: Supplementary file 5 — Supplementary Table S1 [file 41420_2023_1595_MOESM5_ESM.docx]

Supplementary Table S1. Primer sequences used for qRT-PCR.

| Gene symbol | Forward primer sequence（5'to3'） | Reverse primer sequence（5'to3'） |
| --- | --- | --- |
| GAPDH | TGACATCAAGAAGGTGGTGAAGCAG | GTGTCGCTGTTGAAGTCAGAGGAG |
| Cyclin A1 | TTGGGATGGAGACCGGCTTT | CGGGCTGCTGCTGGAA |
| Cyclin A2 | GGACCAGGAGAATATCAACCCG | AAGGGGTGCAACCCGTCTC |
| Cyclin B1 | ACCTGTGTCAGGCTTTCTCTG | CTGACTGCTTGCTCTTCCTCA |
| Cyclin B2 | CTCGGAGAGCAGTCCTAACG | CAAATCACTGGACACCGTCG |
| Cyclin B3 | TGAAAGAGAGAGAGGAACAGTTT | TCTCAAAGGACACCTGCACC |
| Cyclin D1 | CAATGACCCCGCACGATTTC | ATGAACTTCACATCTGTGGCA |
| Cyclin D2 | AGCTGTCACTCCTCATGACT | CATGGCAAACTTAAAGTCGGTG |
| Cyclin D3 | GGTTCCTGTGTCTTTCGCGG | GCTGCTCCTCACATACCCGAG |
| Cyclin E1 | CGCAGGGAGCGGGATG | CTCGCCGTCCTGTCGATTT |
| Cyclin E2 | TAGCTGGTCTGGCGAGGTTT | ATTATCTGGGCTTCTTGGGGG |
| SLC7A11 | TGTGTGGGGTCCTGTCACTA | CAGTAGCTGCAGGGCGTATT |
| GPX4 | AGCAAGATCTGCGTGAACGG | GACGGTGTCCAAACTTGGTG |
